# Association of iron deficiency anaemia with the hospitalization and mortality rate of patients with COVID‑19

**DOI:** 10.3892/mi.2024.193

**Published:** 2024-09-10

**Authors:** Mohammed M. Jalal, Maaidah M. Algamdi, Almohanad A. Alkayyal, Malik A. Altayar, Amr S. Mouminah, Ahlam Jumaa Alamrani, Nouf Abdulaziz Althaqafi, Reem Ali Alamrani, Wjdan Salem Alomrani, Yasmin Attallah Alemrani, Marwan Alhelali, Imadeldin Elfaki, Rashid Mir

**Affiliations:** 1 Department of Medical Laboratory Technology, Prince Fahad Bin Sultan Chair for Biomedical Research, Faculty of Applied Medical Sciences, University of Tabuk, Tabuk 71491, Kingdom of Saudi Arabia; 2 Faculty of Nursing, Community and Mental Health Nursing Department, University of Tabuk, Tabuk 71491, Kingdom of Saudi Arabia; 3 Neuroscience Center, King Abdullah Medical Complex, Jeddah 23816, Kingdom of Saudi Arabia; 4 Department of Statistics, Faculty of Science, University of Tabuk, Tabuk 71491, Kingdom of Saudi Arabia; 5 Department of Biochemistry, Faculty of Science, University of Tabuk, Tabuk 71491, Kingdom of Saudi Arabia

**Keywords:** iron deficiency anaemia, coronavirus disease 2019, hospitalization, Middle East respiratory syndrome coronavirus, severe acute respiratory syndrome coronavirus 2

## Abstract

The severe acute respiratory syndrome coronavirus 2 (SARS-CoV-2) illness led to the coronavirus disease 2019 (COVID-19) pandemic, which has caused enormous health and financial losses, as well as challenges to global health. Iron deficiency anaemia (IDA) has been linked to adverse outcomes in patients infected with SARS-COV-2. The present study aimed to assess the association between IDA and the severity of COVID-19 in hospitalized patients. For this purpose, a retrospective data analysis of 100 patients with COVID-19 was conducted. Data of patients hospitalized with SARS-COV-2 infection confirmed by RT-PCR were collected between June, 2021 and March, 2022. The collected data included patient demographics, comorbidities, clinical signs, symptoms and IDA medical laboratory findings, including complete blood count and iron profiles. The results revealed that patients with COVID-19 admitted to the isolation unit represented 61.0% of the study sample, whereas 39.0% were admitted to the intensive care unit (ICU). No patients had stage I IDA, whereas 4 patients (4%) had stage II IDA. Furthermore, 19 patients (19.0%) had stage III IDA. A significantly higher proportion of patients with IDA (69.6%) were admitted to the ICU compared with those without IDA (29.9%, P<0.001). Additionally, patients with IDA had a higher proportion of a history of stroke compared with those without IDA (17.4 vs. 2.6%, respectively, P=0.024). The most common comorbidities identified were hypertension (29%), diabetes (23%) and heart problems (17%). On the whole, the present study demonstrates significant associations between IDA and a longer hospitalization period. A greater incidence of complications was observed in the hospitalized patients who were SARS-COV-2-positive. Although further studies with larger sample sizes are required to confirm these findings, the results presented herein may provide insight for physicians as regards the prevention and treatment of patients with IDA who are infected with coronavirus.

## Introduction

The severe acute respiratory syndrome coronavirus 2 (SARS-CoV-2) that has caused the continuing coronavirus disease 2019 (COVID-19) pandemic poses a serious threat to health worldwide and the global economy (1). The outbreak of coronavirus originating in December, 2019 was termed SARS-CoV-2 by the World Health Organization (WHO) and the associated disease was named COVID-19(2). By the middle of February 2022, there were over 422 million confirmed cases of COVID-19 confirmed worldwide as per the WHO (3). Moreover, 5.8 million related deaths have occurred due to this disease. Since the time first case of COVID-19 identified in Saudi Arabia on March 2, 2020, the Saudi Ministry of Health has reported 734,389 cases by February 17, 2022, and this included 8,977 related deaths (4,5). Owing to the clinical course of COVD-19 as a subclinical infection, its long incubation period and convalescence, COVID-19 was declared as public health emergency at a global level by the WHO (6). There is a lack of defined treatment protocols for COVID-19, essentially warrants controlling of transmission (7,8). Yet, hospitalization is required in ~20% of symptomatic patients, with 2-5% of patients later requiring management in intensive care units (ICUs) with mechanical ventilation (9,10).

In individuals who are at an advanced age and who have comorbidities, the frequency of hospitalisation, ICU admission and mechanical breathing is markedly higher (11,12,13). Characteristic features of severe infection with COVID-19 include a hyper-inflammatory condition that involves augmented levels of inflammation markers, such as IL-6 and CRP, and increased levels of ferritin (14,15). Ferritin has proven to be the key iron storage protein. Inflammation or iron loading is known to induce the expression of ferritin. In the case of individuals with COVID-19, abnormalities in the concentration of ferritin are associated with the severity of the disease, the development of acute respiratory distress syndrome and mortality (9,14,16). It is well recognised that inflammation alters iron homeostasis. The most common changes include the augmented acquisition of iron, as well as retention inside macrophages and the decreased absorption of iron inside the intestine (17). This leads to a reduced concentration of iron in the circulation and low levels of iron available for erythropoiesis, a process that is required to generate haemoglobin (Hb). In combination with cytokine-mediated erythropoiesis inhibition, the reduced half-life of erythrocytes and diminished erythropoietin activity, this leads to the development of anaemia of inflammation (AI) (18). Low levels of iron and transferrin in the circulation and the diminished saturation of transferrin with iron have been proven to be the characteristic features of AI. However, the ferritin concentration remains at a normal or higher level. Due to this fact, AI differs from iron deficiency anaemia (IDA), which is defined by low ferritin levels, along with elevated transferrin levels (18,19).

Researchers have demonstrated a link between anaemia and worse clinical outcomes of patients with a number of illnesses, including infections and inflammatory diseases (20,21). Reduced levels of iron in the body and increased levels of tissue iron are linked to a reduced lung activity, as well as to severe inflammation of the pulmonary system (22). Anaemia has been proven to be a common manifestation in individuals infected with COVID-19. According to reports, >60% of cases of anaemic COVID-19 have been found to have considerably more adverse outcomes in comparison to COVID-19 cases that were not anaemic (23,24). The WHO has described anaemia as a condition with a Hb level <13 g/dl in males and 12 g/dl in females (25). It has been suggested that the levels of Hb and ferritin are associated with the COVID-19 severity, and that iron metabolism and anaemia affect the pathogenicity, prognosis and treatment of patients with COVID-19 (23,26). Therefore, the present study aimed to examine the association between IDA upon admission to hospital and the outcomes of patients with COVID-19 admitted to King Fahad Specialist Hospital, Tabuk, Kingdom of Saudi Arabia. The present study was conducted in an aim to assess the association between IDA and COVID-19 positivity, as well as severity.

## Patients and methods

### Study design and population

A retrospective data analysis study was conducted at King Fahad Specialist Hospital, Tabuk, Kingdom of Saudi Arabia, which included 100 patients diagnosed with COVID-19 based on the real time-polymerase chain reaction (RT-PCR) confirmation of SARS-COV-2 between June, 2021 and March, 2022. The study followed guidelines from the WHO and the Ministry of Health in the Kingdom of Saudi Arabia for the diagnosis of patients with COVID-19. Outcomes, mortality and length of stay were all reported for all enrolled patients.

### Data collection and sampling

Data for the present study were extracted from the hospital records using a unique medical record number for each patient and were reviewed and examined by a medical team. The extracted data included patient demographics, comorbidities, clinical signs, symptoms, and IDA laboratory findings, including complete blood count and iron profiles. These laboratory examinations were interpreted by the attending physicians. Subjects who tested positive for COVID-19 by RT-PCR in the age group of 18 to 65 years were included in the study; mortal cases due to COVID-19-related complications were also included. Subjects with negative RT-PCR testing for COVID-19, children, subjects with haemorrhagic complications and patients over 65 years old were excluded from the study.

### Ethical considerations

The present study was conducted according to the guidelines outlined in the Declaration of Helsinki and received two ethical approvals from the University of Tabuk Research Ethics Committee (Tabuk, Saudi Arabia; Registration no. UT-198-52-2022) and from the Institutional Review Board General Directorate of Health Affairs, Tabuk Region (Registration no. 612-43-006198). Ethical approvals include agreement for data gathering using data collection forms that include only laboratory results and are organized by unique numbers. No personal Identifier was required, anonymity and confidentiality were maintained as data were accessed and collected by the authors' research team. The need for informed consent for this study was waived by the local ethics research committee, as the present retrospective study using existing data and the subjects cannot be identified directly or indirectly.

### Study variables

The present study assessed the association between IDA and the outcomes of patients with COVID-19 as regards the length of hospitalization and mortality rates. This yielded the following variables: i) COVID-19 mortality rate; ii) COVID-19 hospitalization; iii) IDA.

### Research tools

The authors used four forms to extract data from the patients admitted to the King Fahad Specialist Hospital, Tabuk, Saudi Arabia with a PCR test result confirming SARS-COV-2 infection between June, 2021 and March, 2022. The data were retrieved from the Johns Hopkins patient history form and designated by the authors to fulfil the data required for the study. The data were divided into three forms, as presented in Data S1 (Parts A-C). The first form (Part A) includes the admission data and the outcomes of the medical interventions during the hospitalization period. The second form (Part B) includes data regarding the previous medical history of the participants, including chronic diseases and/or previous major surgeries. The third form (Part C) indicates the laboratory test results, including a PCR swab for COVID-19, complete blood count and iron indices to confirm IDA. The criteria for IDA were adapted from previously published literature (27). The diagnostic criteria for IDA include the following serum markers: Decreased haemoglobin levels (<130 g/l in males; <120 g/l in females; and <110 in pregnant females); a ferritin level of 130 µg/l (no inflammation) and 100 µg/l (in patients with inflammation); the levels of transferrin and the total iron binding capacity are also increased, while the iron level is decreased, the mean corpuscular volume is low, and the transferrin saturations are <20% (27).

### Statistical analysis

Statistical analysis was carried out using RStudio (version 4.1.1) software. Qualitative data are expressed as frequencies and percentages, whereas quantitative data are presented as the median and interquartile range (IQR). The study groups were divided based on the status of IDA. Differences between groups were analysed using Pearson's Chi-squared test or Fisher's exact test for categorical data, whenever applicable. Additionally, the difference in hospitalization period (only) was assessed using the Wilcoxon rank sum test. A value of P<0.05 was considered to indicate a statistically significant difference.

## Results

### Demographic characteristics and patterns of comorbidities

The present study included the records of 100 patients with a confirmed COVID-19 infection. More than half of the patients were females (55.0%) and 41.0% of the patients were aged 45 to <60 years. Patients admitted to the isolation unit represented 61.0% of the sample size, whereas 39.0% of them were admitted to the ICU. The most common comorbidities included hypertension (29.0%), diabetes (23.0%) and heart problems (17.0%) (Table I). Approximately half of the patients had low haemoglobin levels (51.0%) and a low red blood cell (RBC) count (46.0%). The serum iron concentration was low among 40.0% of the patients. The transferrin concentration was low among 42.0% of the patients, and transferrin saturation was low among 41.0% of patients. The characteristics of other blood indices are listed in Table II.

### Characteristics of iron deficiency and the differences between the study groups

The number of patients with IDA was 23 patients (23%). A significantly higher proportion of patients with IDA (69.6%) were admitted to the ICU compared with those without IDA (29.9%; P<0.001). Additionally, the patients with IDA had a higher proportion of a history of stroke compared with those without IDA (17.4 vs. 2.6%; P=0.024) (Table I).

### Presenting symptoms and diagnosis upon admission

The most common presenting symptom among the included patients was cough (25.0%), followed by shortness of breath (20.0%), high blood pressure (12.0%) and fever (10.0%). The rate of cough was significantly lower among patients with IDA than those without IDA (4.3 vs. 31.2%; P=0.009). However, the proportion of patients presenting with high blood pressure was significantly higher among the patients with IDA (26.1 vs. 7.8%; P=0.028) (Table III).

### Outcomes of the patients

The median (IQR) period of hospitalization among the study cohort was 7 days (4 to 15 days), and the patients with IDA had a significantly longer period of hospitalization (median, 15 days; IQR, 8 to 22 days) than those without IDA (median, 5 days; IQR, 3 to 10 days; P<0.001) (Table IV). Generally, 52.0% of the patients were discharged without complications. The proportion of patients who were discharged without complications was significantly lower among the patients with IDA compared with those without IDA (26.1 vs. 59.7%, P=0.005) (Tables IV and V). No significant differences were noted among the IDA and no-IDA cohorts in terms of the rates of other complications, as well as in the rate of mortality (Table V).

## Discussion

It has been demonstrated that >90% of hospitalized patients with COVID-19 have exhibited reduced blood iron levels (28), and that 30% of patients still had iron insufficiency at 60 days following the onset of COVID-19(28). Mutations in the homeostatic iron regulator (*HFE*) gene have been reported to be associated with COVID-19 (29,30). The *HFE* gene encodes the hemochromatosis protein. This protein plays a crucial role in iron metabolism and homeostasis (31).

The present study examined the association between IDA and COVID-19. For this purpose, the data of 100 patients with COVID-19 from the King Fahad Specialist Hospital were collected. Of these 100 patients, 11% patients did not survive, and 61.0 and 39.0% of patients were admitted to the isolation unit and ICU, respectively. Moreover, cardiovascular disorders, diabetes and hypertension were the most prevalent comorbidities among these patients with COVID-19.

IDA is a condition characterized by a reduced concentration of iron. Reduced iron levels may result in the diminished generation of RBCs that transport oxygen to tissues of the body (32). In general, IDA occurs due to inadequate consumption and the absorption of iron and/or loss of this metal due to bleeding (33). Even though the mechanisms through which anaemia augments the susceptibility of an individual to developing infectious disorders remain unclear, and these may involve a number of other factors, such as immunity. IDA affects the health of an individual. In the present study, 23 patients suffered from IDA, indicating an association of IDA with the highest severity of the condition. After analysing the data, it was found that the patients with IDA had greater susceptibility for admission to the ICU in comparison to patients with COVID-19 who did not have IDA. Moreover, the patients with IDA had a longer period of hospitalization. Similarly, a decreased number of patients with IDA were discharged from the hospital without any complications. These results indicated that IDA needs to be considered and treated, particularly in patients suffering from infectious disorders, such as COVID-19.

According to the study conducted by Lechuga *et al* (34), long-term COVID-19 is linked to a number of persistent haematological changes, including changes in RBC composition, anaemia, lymphocytopenia and higher concentrations of inflammatory markers, such as IL-6, D-dimer and ferritin (34). Lechuga *et al* (34) also suggested that the haematological changes associated with long COVID-19 may have implications for the diagnosis, monitoring, and therapy of patients with this disease. Furthermore, Mertens and Peñalvo (35) reported that countries where malnutrition is common and may serve as a driving factor for causing the mortality of patients infected with SARS-CoV-2. Their study reported a moderate positive association between COVID-19 severity and anaemia. They concluded that there was an association between anaemia and an increased risk of developing severe COVID-19(35). Anaemic patients have reduced concentrations of haemoglobin, which transports oxygen to different parts of the body (35). This oxygen transportation is disrupted when the haemoglobin level is low. This usually results in hypoxia, leading to multiple organ dysfunction, including the organs of the respiratory system. This scenario increases the severity of COVID-19, as regards symptoms and outcomes (36). The iron deficiency can result in IDA and increase susceptibility to infection via the suppression of the immune response against pathogens (33,37). The association between iron and infections is not yet fully understood however, and requires further investigations. Previous research has reported that iron deficiency predisposes individuals to infections (38), and studies have also proposed the protective effects of iron (38,39). Therefore, the most efficient strategy to prevent COVID-19 is the maintenance of iron homeostasis (38).

In the present study, the number of patients with a history of stroke was greater in the IDA group in comparison to the non-IDA patients. This result is consistent with the findings of a previous study (40). Chang *et al* (41) investigated the link between stroke and IDA. IDA has been reported to augment the risk of ischemic stroke as it disrupts the transport of oxygen to different tissues via the reduction of haemoglobin (41). In IDA, the secretion of erythropoietin increases the number of RBCs. This secretion stimulates the formation of platelets, leading to thrombocytosis and thrombus formation (42).

In the present study, the results also revealed that the number of patients exhibiting increased blood pressure was also considerably greater in the IDA group as compared with the non-IDA group. This is in line with the study by Savarese *et al* (43), which reported that a deficiency of iron is common in patients with cardiovascular disease (43), and the study by Lanser *et al* (44), which also reported an association of anaemia with cardiovascular disease.

The results of the present study also demonstrated that the patients with IDA had reduced rates of cough as compared with non-IDA COVID-19 patients. The potential association between serum iron and COVID-19 severity was previously investigated by Zhao *et al* (45). They reported that the insufficiency of iron may be one of the factors causing cough in the majority of individuals infected with COVID-19. The result in the present study is in contrast with that in the study by Zhao *et al* (45). The reduced rates of cough among the patients with IDA observed in the present study may be due to the rapid delivery of medical care given to patients at the hospital. Nevertheless, these results are substantially in agreement with those of Zhao *et al* (45), namely that the decreased concentration of serum iron was an independent risk factor for COVID-19.

The present study also demonstrated that IDA was associated with a longer period of hospitalization and with a greater number of complications, eventually necessitating admission into ICUs. Herein, the number of patients with IDA discharged without complications was lower than that of non-IDA patients discharged without complications. It is therefore highly recommended that anaemic patients should be instructed to strongly adhere to the precautionary measures to prevent the infections. Moreover, physicians should closely monitor anaemic patients to avoid adverse outcomes due to COVID-19. Anaemia may be considered one of the factors in the development of risk stratification models for COVID-19 in the future.

The present study had certain limitations, which should be mentioned. The present study was a single-centre study with a limited sample size. The association between IDA and COVID-19 needs to be further be investigated in multicentre, larger-scales studies to verify the findings presented herein.

In conclusion, the present study demonstrated significant associations between IDA and longer periods of hospitalization, and a greater incidence of complications in hospitalised patients with SARS-COV-2. However, the results should be interpreted with caution, given the retrospective nature of the study and the constraints on data collection. Considering the notable effect of IDA on different aspects of COVID-19 revealed in the present study, screening tests need to be carried out to identify patients with IDA followed by the treatment of IDA to improve patient outcomes. IDA can be treated through blood transfusions, the administration of iron supplements or other strategies. Moreover, different measures need to be taken to prevent and treat cases with IDA against viral infection.

## Supplementary Material

Supplementary Data.

## Figures and Tables

**Table I:** Characteristics of the demographic data and comorbidities among the overall cohort and among patients with and without IDA.

			IDA		
Parameter	Category	Overall, n=100 (%)	No, n=77 (%)	Yes, n=23 (%)	P-value
Age, years	18 to <30	21 (21.0)	18 (23.4)	3 (13.0)	0.340
	30 to <45	19 (19.0)	16 (20.8)	3 (13.0)	
	45 to <60	41 (41.0)	31 (40.3)	10 (43.5)	
	≥60	19 (19.0)	12 (15.6)	7 (30.4)	
Sex	Male	45 (45.0)	36 (46.8)	9 (39.1)	0.519
	Female	55 (55.0)	41 (53.2)	14 (60.9)	
Smoking	No	63 (63.0)	48 (62.3)	15 (65.2)	>0.999
	Yes	24 (24.0)	19 (24.7)	5 (21.7)	
	Do not know	13 (13.0)	10 (13.0)	3 (13.0)	
Unit patient admitted to	Isolation unit	61 (61.0)	54 (70.1)	7 (30.4)	**<0.001**
	ICU	39 (39.0)	23 (29.9)	16 (69.6)	
Comorbidities	Non-IDA anaemia	6 (6.0)	5 (6.5)	1 (4.3)	>0.999
	Angina	5 (5.0)	3 (3.9)	2 (8.7)	0.324
	Heart problems	17 (17.0)	10 (13.0)	7 (30.4)	0.063
	Asthma	10 (10.0)	10 (13.0)	0 (0.0)	0.111
	Cancer	4 (4.0)	3 (3.9)	1 (4.3)	>0.999
	Stomach or peptic ulcer	1 (1.0)	1 (1.3)	0 (0.0)	>0.999
	Hypertension	29 (29.0)	19 (24.7)	10 (43.5)	0.081
	COPD	1 (1.0)	1 (1.3)	0 (0.0)	>0.999
	Diabetes	23 (23.0)	16 (20.8)	7 (30.4)	0.334
	ARF type 2	1 (1.0)	1 (1.3)	0 (0.0)	>0.999
	Epilepsy	3 (3.0)	2 (2.6)	1 (4.3)	0.548
	OSA	1 (1.0)	1 (1.3)	0 (0.0)	>0.999
	kidney disease	3 (3.0)	3 (3.9)	0 (0.0)	>0.999
	Lung fibrosis	1 (1.0)	0 (0.0)	1 (4.3)	0.230
	High cholesterol	7 (7.0)	5 (6.5)	2 (8.7)	0.659
	Surgery	11 (11.0)	6 (7.8)	5 (21.7)	0.120
	Pneumonia	10 (10.0)	7 (9.1)	3 (13.0)	0.692
	Stroke	6 (6.0)	2 (2.6)	4 (17.4)	**0.024**
	Mature cataract	1 (1.0)	1 (1.3)	0 (0.0)	>0.999
	ARDS	1 (1.0)	1 (1.3)	0 (0.0)	>0.999
	Leukaemia	1 (1.0)	0 (0.0)	1 (4.3)	0.230
	Hereditary sensory motor neuropathy	1 (1.0)	0 (0.0)	1 (4.3)	0.230
	MV REGURG	1 (1.0)	0 (0.0)	1 (4.3)	0.230

Data were analysed using the Chi-squared test or Fisher's exact test. Values in bold font indicate statistically significant differences (P<0.05). IDA, iron deficiency anaemia; ICU, intensive care unit; COPD, chronic obstructive pulmonary disease; ARF, acute respiratory failure; OSA, obstructive sleep apnoea; ARDS, acute respiratory distress syndrome; MV REGURG, mitral valve regurgitation.

**Table II:** Characteristics of blood and iron profiles among the included patients.

Parameter	Category	n (%)
Haemoglobin	Normal	33 (33.0)
	Low	51 (51.0)
	High	16 (16.0)
RBC count	Normal	43 (43.0)
	Low	46 (46.0)
	High	11 (11.0)
WBC count	Normal	33 (33.0)
	Low	5 (5.0)
	High	62 (62.0)
Platelet count	Normal	58 (58.0)
	Low	15 (15.0)
	High	27 (27.0)
MCV	Normal	52 (52.0)
	Low	39 (39.0)
	High	9 (9.0)
MCH	Normal	50 (50.0)
	Low	45 (45.0)
	High	5 (5.0)
MCHC	Normal	53 (53.0)
	Low	46 (46.0)
	High	1 (1.0)
Serum iron	Normal	54 (54.0)
	Low	40 (40.0)
	High	6 (6.0)
Transferrin	Normal	55 (55.0)
	Low	42 (42.0)
	High	3 (3.0)
Ferritin	Normal	35 (35.0)
	Low	28 (28.0)
	High	37 (37.0)
TIBC	Normal	54 (54.0)
	Low	15 (15.0)
	High	31 (31.0)
Transferrin saturation (%)	Normal	56 (56.0)
	Low	41 (41.0)
	High	3 (3.0)

RBC, red blood cell; WBC, white blood cell; MCV, mean corpuscular volume; MCH, mean corpuscular haemoglobin; MCHC, mean corpuscular haemoglobin concentration; TIBC, total iron-binding capacity.

**Table III:** Presenting symptoms and diagnosis of upon admission among the overall cohort and among patients with and without IDA.

		IDA		
Parameter	Overall, n=100 (%)	No, n=77 (%)	Yes, n=23 (%)	P-value
Cough	25 (25.0)	24 (31.2)	1 (4.3)	**0.009**
SOB	20 (20.0)	14 (18.2)	6 (26.1)	0.391
High blood pressure	12 (12.0)	6 (7.8)	6 (26.1)	**0.028**
Fever	10 (10.0)	9 (11.7)	1 (4.3)	0.446
COVID-19 (mortality)	11 (11.0)	9 (11.7)	2 (8.7)	>0.999
Asthma	8 (8.0)	8 (10.4)	0 (0.0)	0.192
Chest pain	7 (7.0)	6 (7.8)	1 (4.3)	>0.999
Pneumonia	5 (5.0)	3 (3.9)	2 (8.7)	0.324
ARDS	3 (3.0)	2 (2.6)	1 (4.3)	0.548
Nausea	1 (1.0)	1 (1.3)	0 (0.0)	>0.999
Loss of appetite	1 (1.0)	1 (1.3)	0 (0.0)	>0.999
Acute bronchitis	1 (1.0)	0 (0.0)	1 (4.3)	0.230
Dyspnoea	1 (1.0)	1 (1.3)	0 (0.0)	>0.999
Oesophageal varices bleeding	1 (1.0)	0 (0.0)	1 (4.3)	0.230
CVA	1 (1.0)	1 (1.3)	0 (0.0)	>0.999
Renal failure	1 (1.0)	1 (1.3)	0 (0.0)	>0.999
Myocardial infarction	1 (1.0)	1 (1.3)	0 (0.0)	>0.999
COPD	1 (1.0)	1 (1.3)	0 (0.0)	>0.999
Mature cataract	1 (1.0)	1 (1.3)	0 (0.0)	>0.999
Thrombosis	1 (1.0)	0 (0.0)	1 (4.3)	0.230
AF	1 (1.0)	0 (0.0)	1 (4.3)	0.230
Stroke	1 (1.0)	0 (0.0)	1 (4.3)	0.230
Ischemic cardiomyopathy	1 (1.0)	0 (0.0)	1 (4.3)	0.230
Anal fistula	1 (1.0)	0 (0.0)	1 (4.3)	0.230
Acute respiratory failure type 2	1 (1.0)	1 (1.3)	0 (0.0)	>0.999
CAD	1 (1.0)	1 (1.3)	0 (0.0)	>0.999

Data were analysed using the Chi-squared test or Fisher's exact test. Values in bold font indicate statistically significant differences (P<0.05). IDA, iron deficiency anaemia; COPD, chronic obstructive pulmonary disease; SOB, shortness of breath; ARDS, acute respiratory distress syndrome; CVA, cerebral vascular accident; AF, atrial fibrillation; CAD, coronary artery disease.

**Table IV:** Outcomes of the overall cohort and patients with and without IDA.

		IDA		
Parameter	Overall, n=100	No, n=77	Yes, n=23	P-value
Hospitalization period (days)^a^	7 (4-15)	5 (3-10)	15 (8-22)	**<0.001**
Discharged with no complications	52 (52.0%)	46 (59.7%)	6 (26.1%)	**0.005**
ARDS	1 (1.0%)	1 (1.3%)	0 (0.0%)	>0.999
Pneumonia	4 (4.0%)	3 (3.9%)	1 (4.3%)	>0.999
Septic shock	3 (3.0%)	1 (1.3%)	2 (8.7%)	0.131
Multi-organ failure	2 (2.0%)	1 (1.3%)	1 (4.3%)	0.409
Death	11 (11.0%)	7 (9.1%)	4 (17.4%)	0.271

^a^Results are expressed as the median (interquartile range); otherwise, the results are presented as frequencies and percentages. Data were analysed using the Wilcoxon rank-sum test, Chi-squared test, or Fisher's exact test. Values in bold font indicate statistically significant differences (P<0.05). IDA, iron deficiency anaemia; ARDS, acute respiratory distress syndrome.

**Table V:** Outcomes of patients with and without IDA.

		IDA		
Parameter	Overall, n=100 (%)	No, n=77 (%)	Yes, n=23 (%)	P-value
Mortality	11 (11.0)	7 (9.1)	4 (17.4)	0.271
ICU	39 (39.0)	23 (29.9)	16 (69.6)	**<0.001**
Discharged with no complications	52 (52.0)	46 (59.7)	6 (26.1)	**0.005**
Suffering from complications	37 (37.0)	25 (32.5)	12 (52.2)	0.086

Data were analysed using the Chi-squared test or Fisher's exact test. Values in bold font indicate statistically significant differences (P<0.05). IDA, iron deficiency anaemia; ICU, intensive care unit.

## Data Availability

The datasets used and/or analysed during the current study are available from the corresponding author on reasonable request.

## References

[1] Yin Y, Wunderink RG (2018). MERS, SARS and other coronaviruses as causes of pneumonia. Respirology.

[2] Peng PWH, Ho PL, Hota SS (2020). Outbreak of a new coronavirus: What anaesthetists should know. Br J Anaesth.

[3] https://www.who.int/publications/m/item/weekly-epidemiological-update-on-covid-19-22-february-2022.

[4] Nurunnabi M (2021). The preventive strategies of COVID-19 pandemic in Saudi Arabia. J Microbiol Immunol Infect.

[5] Salam AA, Al-Khraif RM, Elsegaey I (2021). COVID-19 in saudi arabia: An overview. Front Public Health.

[6] https://www.who.int/publications/m/item/covid-19-public-health-emergency-of-international-concern-(pheic)-global-research-and-innovation-forum.

[7] Singhal T (2020). A review of coronavirus Disease-2019 (COVID-19). Indian J Pediatr.

[8] Smatti MK, Al Thani AA, Yassine HM (2018). Viral-induced enhanced disease illness. Front Microbiol.

[9] Wu Z, McGoogan JM (2020). Characteristics of and important lessons from the coronavirus disease 2019 (COVID-19) outbreak in China: Summary of a report of 72 314 cases from the Chinese center for disease control and prevention. JAMA.

[10] Bi Q, Wu Y, Mei S, Ye C, Zou X, Zhang Z, Liu X, Wei L, Truelove SA, Zhang T (2020). Epidemiology and transmission of COVID-19 in 391 cases and 1286 of their close contacts in Shenzhen, China: A retrospective cohort study. Lancet Infect Dis.

[11] Murthy S, Gomersall CD, Fowler RA (2020). Care for Critically Ill patients With COVID-19. JAMA.

[12] Yang J, Zheng Y, Gou X, Pu K, Chen Z, Guo Q, Ji R, Wang H, Wang Y, Zhou Y (2020). Prevalence of comorbidities and its effects in patients infected with SARS-CoV-2: A systematic review and meta-analysis. Int J Infect Dis.

[13] Lippi G, Henry BM (2020). Chronic obstructive pulmonary disease is associated with severe coronavirus disease 2019 (COVID-19). Respir Med.

[14] Zhou F, Yu T, Du R, Fan G, Liu Y, Liu Z, Xiang J, Wang Y, Song B, Gu X (2020). Clinical course and risk factors for mortality of adult inpatients with COVID-19 in Wuhan, China: A retrospective cohort study. Lancet.

[15] Herold T, Jurinovic V, Arnreich C, Lipworth BJ, Hellmuth JC, von Bergwelt-Baildon M, Klein M, Weinberger T (2020). Elevated levels of IL-6 and CRP predict the need for mechanical ventilation in COVID-19. J Allergy Clin Immunol.

[16] Henry BM, de Oliveira MHS, Benoit S, Plebani M, Lippi G (2020). Hematologic, biochemical and immune biomarker abnormalities associated with severe illness and mortality in coronavirus disease 2019 (COVID-19): A meta-analysis. Clin Chem Lab Med.

[17] Theurl I, Aigner E, Theurl M, Nairz M, Seifert M, Schroll A, Sonnweber T, Eberwein L, Witcher DR, Murphy AT (2009). Regulation of iron homeostasis in anemia of chronic disease and iron deficiency anemia: Diagnostic and therapeutic implications. Blood.

[18] Weiss G, Ganz T, Goodnough LT (2019). Anemia of inflammation. Blood.

[19] Camaschella C (2019). Iron deficiency. Blood.

[20] Cappellini MD, Comin-Colet J, de Francisco A, Dignass A, Doehner W, Lam CS, Macdougall IC, Rogler G, Camaschella C, Kadir R (2017). Iron deficiency across chronic inflammatory conditions: International expert opinion on definition, diagnosis, and management. Am J Hematol.

[21] Wouters H, van der Klauw MM, de Witte T, Stauder R, Swinkels DW, Wolffenbuttel BHR, Huls G (2019). Association of anemia with health-related quality of life and survival: A large population-based cohort study. Haematologica.

[22] Brigham EP, McCormack MC, Takemoto CM, Matsui EC (2015). Iron status is associated with asthma and lung function in US women. PLoS One.

[23] Bergamaschi G, Borrelli de Andreis F, Aronico N, Lenti MV, Barteselli C, Merli S, Pellegrino I, Coppola L, Cremonte EM, Croce G (2021). Anemia in patients with Covid-19: Pathogenesis and clinical significance. Clin Exp Med.

[24] Faghih Dinevari M, Somi MH, Sadeghi Majd E, Abbasalizad Farhangi M, Nikniaz Z (2021). Anemia predicts poor outcomes of COVID-19 in hospitalized patients: A prospective study in Iran. BMC Infect Dis.

[25] McLean E, Cogswell M, Egli I, Wojdyla D, de Benoist B (2009). Worldwide prevalence of anaemia, WHO Vitamin and Mineral Nutrition Information System, 1993-2005. Public Health Nutr.

[26] Taneri PE, Gomez-Ochoa SA, Llanaj E, Raguindin PF, Rojas LZ, Roa-Diaz ZM, Salvador D Jr, Groothof D, Minder B, Kopp-Heim D (2020). Anemia and iron metabolism in COVID-19: A systematic review and meta-analysis. Eur J Epidemiol.

[27] Kumar A, Sharma E, Marley A, Samaan MA, Brookes MJ (2022). Iron deficiency anaemia: Pathophysiology, assessment, practical management. BMJ Open Gastroenterol.

[28] Suriawinata E, Mehta KJ (2023). Iron and iron-related proteins in COVID-19. Clin Exp Med.

[29] Hubacek JA, Philipp T, Adamkova V, Majek O, Dusek L (2023). A haemochromatosis-causing HFE mutation is associated with SARS-CoV-2 susceptibility in the Czech population. Clin Chim Acta.

[30] Mir R, Elfaki I, Alanazi MA, Algehainy NA, Altemani FH, Alsayed BA, Mohamed EI, Mustafa SK, Moawadh MS, Tayeb FJ (2024). Whole-Exome sequencing detecting a recurrent pathogenic mutation, HFE p.His63Asp (H63D) in COVID-19 patients and its effect on mortality. Discov Med.

[31] Reuben A, Chung JW, Lapointe R, Santos MM (2017). The hemochromatosis protein HFE 20 years later: An emerging role in antigen presentation and in the immune system. Immun Inflamm Dis.

[32] Zhou ZD, Tan EK (2017). Iron regulatory protein (IRP)-iron responsive element (IRE) signaling pathway in human neurodegenerative diseases. Mol Neurodegener.

[33] Jonker FA, Boele van Hensbroek M (2014). Anaemia, iron deficiency and susceptibility to infections. J Infect.

[34] Lechuga GC, Morel CM, De-Simone SG (2023). Hematological alterations associated with long COVID-19. Front Physiol.

[35] Mertens E, Penalvo JL (2020). The Burden of Malnutrition and Fatal COVID-19: A global burden of disease analysis. Front Nutr.

[36] Hariyanto TI, Kurniawan A (2020). Anemia is associated with severe coronavirus disease 2019 (COVID-19) infection. Transfus Apher Sci.

[37] Aslan ES, Aydin H, Tekin YK, Keles S, White KN, Hekim N (2023). Association between iron metabolism and SARS-COV-2 infection, determined by ferritin, hephaestin and hypoxia-induced factor-1 alpha levels in COVID-19 patients. Mol Biol Rep.

[38] Morais AHA, Aquino JS, da Silva-Maia JK, Vale SHL, Maciel BLL, Passos TS (2021). Nutritional status, diet and viral respiratory infections: Perspectives for severe acute respiratory syndrome coronavirus 2. Br J Nutr.

[39] Menshawey R, Menshawey E, Alserr AHK, Abdelmassih AF (2020). Low iron mitigates viral survival: Insights from evolution, genetics, and pandemics-a review of current hypothesis. Egypt J Med Hum Genet.

[40] Desai A, Oh D, Rao EM, Sahoo S, Mahajan UV, Labak CM, Mauria R, Shah VS, Nguyen Q, Herring EZ (2023). Impact of anemia on acute ischemic stroke outcomes: A systematic review of the literature. PLoS One.

[41] Chang YL, Hung SH, Ling W, Lin HC, Li HC, Chung SD (2013). Association between ischemic stroke and iron-deficiency anemia: A population-based study. PLoS One.

[42] Heo J, Youk TM, Seo KD (2021). Anemia is a risk factor for the development of ischemic stroke and Post-Stroke mortality. J Clin Med.

[43] Savarese G, von Haehling S, Butler J, Cleland JGF, Ponikowski P, Anker SD (2023). Iron deficiency and cardiovascular disease. Eur Heart J.

[44] Lanser L, Fuchs D, Scharnagl H, Grammer T, Kleber ME, März W, Weiss G, Kurz K (2021). Anemia of chronic disease in patients with cardiovascular disease. Front Cardiovasc Med.

[45] Zhao K, Huang J, Dai D, Feng Y, Liu L, Nie S (2020). Serum iron level as a potential predictor of coronavirus disease 2019 severity and mortality: A retrospective study. Open Forum Infect Dis.

