# Supplementary material for: Association of iron deficiency anaemia with the hospitalization and mortality rate of patients with COVID‑19
Source: Med Int (Lond). 2024 Sep 10;4(6):69. doi: 10.3892/mi.2024.193 (PMC11411605; doi:10.3892/mi.2024.193)
Supplement: Supplementary Data. [file Supplementary_Data.pdf]

Data S1.

Part A: The first form

Admission date: ___ / ____ / 2022
Age: ___________
Sex: ❑Male ❑Female
Medical diagnosis: ________________________
Smoking: ❑yes ❑No

Outcomes:

❑ **Discharged with no complications**

❑ **Suffer from complications**

- ❑ Pneumonia
- ❑ Acute respiratory distress syndrome (ARDS)
- ❑ Multi-organ failure
- ❑ Septic shock
- ❑ Others: _______________________
- ❑ Death

Discharge date: ___ / ____ / 2022

Part B: The second form

PAST MEDICAL HISTORY

Are there any previous conditions chronic and/or surgeries?

- ❑ Diabetes (Type _______)
- ❑ Crohn's disease
- ❑ Pneumonia
- ❑ High cholesterol
- ❑ Anemia
- ❑ Asthma
- ❑ Goiter
- ❑ Hepatitis
- ❑ Stroke
- ❑ Leukemia
- ❑ Rheumatic fever
- ❑ Cataracts
- ❑ Angina
- ❑ HIV/AIDS
- ❑ Kidney stones
- ❑ Heart murmur
- ❑ High blood pressure
- ❑ Colitis
- ❑ Pulmonary embolism
- ❑ Hypothyroidism
- ❑ Jaundice
- ❑ Emphysema
- ❑ Cancer (Type: ____________)
- ❑ Stomach or peptic ulcer
- ❑ Epilepsy (seizures)
- ❑ Psoriasis
- ❑ Tuberculosis
- ❑ kidney disease
- ❑ Heart problems
- ❑ Surgery: _____________

Part C: The third form

Laboratory test results<br>PCR swab for COVID-19:	❑+ve	❑-ve
Hgb		❑Normal<br>❑High<br>❑Low
RBCs count		❑Normal<br>❑High<br>❑Low
WBCs count		❑Normal<br>❑High<br>❑Low
PLT count		❑Normal<br>❑High<br>❑Low

MCV		❑Normal<br>❑High<br>❑Low
MCH		❑Normal<br>❑High<br>❑Low
MCHC		❑Normal<br>❑High<br>❑Low
Serum iron		❑Normal<br>❑High<br>❑Low
Transferrin		❑Normal<br>❑High<br>❑Low
Ferritin		❑Normal<br>❑High<br>❑Low
Total iron-binding capacity (TIBC)		❑Normal<br>❑High<br>❑Low
Transferrin saturation (%)		❑Normal<br>❑High<br>❑Low
